# Menopausal symptoms are associated with oral sensory complaints in perimenopausal women: an observational study

**DOI:** 10.1186/s12905-021-01401-6

**Published:** 2021-06-30

**Authors:** Tomoharu Taga, Kayoko Ito, Kiyoshi Takamatsu, Mariko Ogawa, Saori Funayama, Makoto Inoue

**Affiliations:** 1 Division of Dysphagia Rehabilitation, Niigata University Graduate School of Medical and Dental Sciences, Niigata, Japan; 2 Dental Oral and Maxillofacial Surgery, Fukui Prefectural Hospital, Fukui, Japan; 3 Oral Rehabilitation, Niigata University Medical and Dental Hospital, 1-754 Asahimachi-dori, Chuou-ku, Niigata, 951-8520 Japan; 4 Department of Obstetrics and Gynecology, Tokyo Dental College Ichikawa General Hospital, Chiba, Japan

**Keywords:** Oral sensory complaint, Xerostomia, Taste disturbance, Burning mouth, Menopause

## Abstract

**Background:**

Perimenopausal women experience a wide variety of systemic symptoms: hot flashes, sweating, mental health concerns and various oral sensory complaints (OSC). OSC in perimenopausal women include xerostomia, taste disturbance and burning mouth. However, the factors associated with these OSC have not been identified. The purpose of this investigation was to elucidate the factors associated with OSC in perimenopausal women.

**Methods:**

The study cohort comprised 43 perimenopausal women aged 45–55 years. Data on medical history, medications, menstrual status, menopausal symptoms, quality of life, xerostomia, taste disturbance and burning mouth were collected. Volumes of unstimulated and stimulated saliva were measured. Tongue coating was evaluated according to a tongue coating index. Univariate analysis was performed to identify factors significantly associated with having xerostomia, taste disturbance, burning mouth and more than two OSC (2OSC). Next, the factors strongly associated with these symptoms were examined by logistic regression analysis.

**Results:**

The number of menopausal symptoms was significantly higher, and volume of unstimulated saliva was significantly lower in participants with xerostomia, taste disturbance, burning mouth or 2OSC than in those without these characteristics. Agents targeting the central nervous system were more frequently taken by participants with burning mouth and 2OSC than by those without these characteristics. According to logistic regression analysis, the number of menopausal symptoms was an explanatory variable for xerostomia, taste disturbance, burning mouth and 2OSC.

**Conclusions:**

Our findings suggested that OSC associated with the number of menopausal symptoms. Management of menopausal symptoms may decrease OSC, leading to improved quality of life of perimenopausal women.

**Supplementary Information:**

The online version contains supplementary material available at 10.1186/s12905-021-01401-6.

## Background

During perimenopausal period, many women experience a wide variety of systemic symptoms, including symptoms such as hot flashes, sweating, mental health concerns, and mucocutaneous symptoms, all of which can markedly decrease their quality of life (QOL). These symptoms are mainly attributable to a decline in production of female sex hormones. Various oral sensory disturbances, including xerostomia, taste disturbance and burning mouth have also been reported [1]. These sensory disturbances have been classified as oral sensory complaints (OSC) [2, 3].

Xerostomia is defined as a subjective experience of a dry mouth [4], whereas hyposalivation is an objective reduction in salivary secretion [5]. Causes of xerostomia vary widely, including Sjögren syndrome, psychological stress, radiation therapy, medications and salivary gland tumors. An association between xerostomia and menopausal status has been widely reported [1, 6]. Burning mouth syndrome is defined by the International Association for the Study of Pain as a burning pain in the tongue and/or other oral mucous membranes in the absence of clinical signs and abnormal laboratory findings [7]. Burning mouth syndrome occurs in 10–40% of women of perimenopausal age, decreasing their QOL [8]. Indeed, in one study 79.5% of physicians and gynecologists reported encountering the complaints xerostomia, taste disturbance, burning mouth or temporomandibular disorders [9]. After ascertaining the presence of these symptoms, 58.5% of these physicians reported referring affected women to specialized clinics. In other words, the remaining women potentially failed to receive appropriate medical care.

If specific menopausal symptoms, or the number of such symptoms, are associated with OSC in perimenopausal women, treatment of those symptoms would likely contribute to improving their QOL. However, to our knowledge, no reported studies have investigated the factors associated with OSC in individual perimenopausal women. The purpose of this study was to identify those factors.

## Subjects and methods

### Participants

Before the study was initiated, a required sample size of 43 was calculated using software (G*power 3.1.9.7) and an effect size of 0.5, α-value of 0.05, and power of 0.9. Inclusion criteria were: women aged 45 to 55 years. Exclusion criteria were: pregnant women or women who had undergone previous hysterectomy or bilateral oophorectomy. The participants were drawn from patients and staff of the departments of Oral Surgery in Fukui Prefectural Hospital and Oral Rehabilitation in Niigata Medical and Dental Hospital in Japan between 2018 and 2020.

### Questionnaires

#### Characteristics of participants and oral symptoms

Each study participant filled out questionnaires that included items on age, medical history, medications, menstrual status, menopausal symptoms, history of treatment for menopausal symptoms, oral symptoms including xerostomia, taste disturbance and burning mouth (Additional file 1: Supplementary table 1). Reported medication history was confirmed by checking the individual’s medical records. Package inserts were examined to determine whether the reported medications had the adverse effects of xerostomia, taste disturbance or burning mouth.

#### Menopausal symptoms

To assess menopausal symptoms, we used the menopausal symptom checklist for Japanese women [10], which includes 21 items (Additional file 2: Supplementary table 2). These items are allotted scores on a three-point scale (2: severe, 1: mild, 0: none). This checklist was developed for Japanese women [10] whereas the Kuppermann index was developed for American women [11]. We evaluated xerostomia, taste disturbance and burning mouth using the same three-point scale.

#### QOL

The Japanese version of the 36-Item Short-Form Health (SF-36) [12] was used to assess QOL. SF-36 includes one multi-item scale that assesses eight health concepts: (1) limitations in physical activities because of health problems; (2) limitations in social activities because of physical or emotional problems; (3) limitations in usual role activities because of physical health problems; (4) bodily pain; (5) general mental health (psychological distress and well-being); (6) limitations in usual role activities because of emotional problems; (7) vitality (energy and fatigue); and (8) general health perceptions [12].

#### Volume of saliva and tongue coating

All participants were asked not to drink or eat for at least 1 h before assessment. We measured the volume of unstimulated saliva using the 10-min spitting method; 1 g of saliva was converted to 1 mL [13]. Stimulated saliva volume was measured by the Saxon method [14]. Tongue coating was evaluated with the Tongue Coating Index (TCI) [15], which entails evaluating the thickness of tongue coating in nine sections of the tongue surface (Score 0: no tongue coating visible; Score 1: tongue coating thin, papillae of tongue visible; Score 2: tongue coating thick, papillae of tongue not visible). The TCI is obtained by dividing the total score by 18 and then multiplying by 100.

### Statistical analysis

Participants with self-reported mild-to-severe xerostomia, taste disturbance or burning mouth were considered to have the relevant symptom. Participants with two or more of these symptoms were classified as having two or more OSC (2OSC). The presence of mild-to-severe menopausal symptoms was determined on the basis of self-reporting. We used a three-component summary score (physical, mental, and role/social component summaries) to classify responses to the SF-36 questionnaire.

We performed univariate analysis to investigate the relationship between xerostomia, taste disturbance, burning mouth, and 2OSC and the other evaluated characteristics. The Mann–Whitney U test was performed for numerical data because all data were non-normally distributed. χ^2^ test was performed for categorical data. Logistic regression analysis using a stepwise method was employed to assess factors associated with xerostomia, taste disturbance, burning mouth, and 2OSC. The presence of xerostomia was treated as an objective variable, whereas variables showing significant associations by the χ^2^ and Mann–Whitney U tests were treated as explanatory variables. In choosing explanatory variables, we considered the correlations with each factor and selected only one of the items with a Spearman’s correlation coefficient of 0.7 or more. The statistical software used was SPSS 26.0 (IBM, Japan). Statistical significance was set at *P* < 0.05.

## Results

Forty-three 45–55-year-old women participated in this survey and all of them completed the questionnaires and measurement of volume of saliva and tongue coatings.

### Oral sensory complains and characteristics

Characteristics of the participants are shown in the left-hand column of Table 1. Of the 43 participants, 22 (51.2%) had xerostomia, 12 (27.9%) had taste disturbance, 13 (30.2%) had burning mouth and 15 (34.9%) had 2OSC. The results of univariate analysis are shown in Table 1. Women with taste disturbance and 2OSC reported taking significantly more medications than did the other participants (*p* = 0.04 and *p* < 0.001, respectively). Agents targeting the central nervous system were more frequently taken by participants with burning mouth and 2OSC than by those without these characteristics (*p* = 0.014 and *p* = 0.003, respectively).

**Table 1:** Factors associated with having xerostomia, taste disturbance, burning mouth and two or more oral sensory complaints (univariate analysis)

		Total	Xerostomia			Taste disturbance			Burning mouth			2OSC		
		(n = 43)	no (n = 21)	yes (n = 22)	*p* value	no (n = 31)	yes (n = 12)	*p* value	no (n = 30)	yes (n = 13)	*p* value	no (n = 28)	yes (n = 15)	*p* value
*Characteristics*														
Age	mean ± SD	48.7 ± 4.7	49.2 ± 3	48.3 ± 5.8	0.92	48.9 ± 5.2	48.3 ± 2.9	0.25	48.5 ± 5.1	49.2 ± 3.4	0.89	48.5 ± 5.4	49.3 ± 2.8	0.86
BMI	mean ± SD	22.3 ± 3.7	22.6 ± 3.6	21.9 ± 3.9	0.34	21.9 ± 3.9	23.2 ± 3.4	0.17	21.6 ± 3.2	23.8 ± 4.5	0.08	21.6 ± 3.4	23.5 ± 4.2	0.13
Married	n (%)	31 (72.1)	17 (81.0)	14 (63.6)	0.21	23 (74.2)	8 (66.7)	0.45	23 (76.7)	8 (61.5)	0.26	22 (78.6)	9 (60.0)	0.17
Children	n (%)	12 (27.9)	4 (19.0)	8 (36.4)	0.21	8 (25.8)	4 (33.3)	0.45	6 (20.0)	6 (46.2)	0.09	6 (21.4)	6 (40.0)	0.17
Job	n (%)	36 (83.7)	19 (90.5)	17 (77.3)	0.23	25 (80.6)	11 (91.7)	0.36	25 (83.3)	11 (84.6)	0.65	24 (85.7)	12 (80.0)	0.47
Smoking	n (%)	1 (2.3)	0 (0.0)	1 (4.5)	0.51	1 (3.2)	0 (0.0)	0.72	0 (0.0)	1 (7.7)	0.30	0 (0.0)	1 (6.7)	0.35
*Medical history*														
Hypertension	n (%)	5 (11.6)	3 (14.3)	2 (9.1)	0.48	4 (12.9)	1 (8.3)	0.57	3 (10.0)	2 (15.4)	0.48	3 (10.7)	2 (13.3)	0.58
Psychosis	n (%)	5 (11.6)	1 (4.8)	4 (18.2)	0.19	3 (9.7)	2 (16.7)	0.43	1 (3.3)	4 (30.8)	0.02	1 (3.6)	4 (26.7)	0.04
*Medication*														
Number of drugs	mean ± SD	2.2 ± 4.4	0.7 ± 1.1	3.6 ± 5.7	0.07	1.5 ± 3.2	3.9 ± 6.3	0.04	1.3 ± 3.1	4.1 ± 6.2	0.04	0.8 ± 1.8	4.7 ± 6.4	< 0.001
CNS agent	n (%)	7 (16.3)	2 (9.5)	5 (23.8)	0.21	3 (10.0)	4 (33.3)	0.09	2 (6.7)	5 (41.7)	0.01	1 (3.6)	6 (42.9)	0.00
Antihypertensive	n (%)	3 (7.0)	1 (4.8)	2 (9.5)	0.50	2 (6.7)	1 (8.3)	0.65	1 (3.3)	2 (16.7)	0.19	1 (3.6)	2 (14.3)	0.25
*Adverse effect of medication*														
Xerostomia	n (%)	15 (34.9)	7 (33.3)	8 (38.1)	0.75	8 (26.7)	7 (58.3)	0.06	8 (26.7)	7 (58.3)	0.06	6 (21.4)	9 (64.3)	0.01
Taste disturbance	n (%)	10 (23.3)	5 (23.8)	5 (23.8)	1.00	8 (26.7)	2 (16.7)	0.4	7 (23.3)	3 (25.0)	0.60	6 (21.4)	4 (28.6)	0.44
Burning mouth	n (%)	1 (2.3)	0 (0.0)	1 (4.8)	0.31	0 (0.0)	1 (8.3)	0.29	1 (3.3)	0 (0.0)	0.71	0 (0.0)	1 (7.1)	0.33
*Sleep*														
Sleeping duration (min)	mean ± SD	356.7 ± 82.7	346.2 ± 55.5	366.8 ± 102.6	0.39	349.4 ± 88.5	375.8 ± 64.9	0.17	348 ± 85.8	376.9 ± 74.3	0.08	349.3 ± 84.1	370.7 ± 80.9	0.11
Lach of sleeping time	n (%)	18 (41.9)	8 (38.1)	10 (45.5)	0.62	13 (41.9)	5 (41.7)	0.99	14 (46.7)	4 (30.8)	0.33	12 (42.9)	6 (40.0)	0.86
Quarity of sleeping	n (%)	19 (44.2)	8 (38.1)	11 (50.0)	0.43	13 (41.9)	6 (50.0)	0.63	13 (43.3)	6 (46.2)	0.86	12 (42.9)	7 (46.7)	0.81
*Quarity of life*														
SDS total	mean ± SD	42.3 ± 9.7	38.9 ± 8.6	45.6 ± 9.7	0.05	41.4 ± 9.5	44.7 ± 10.3	0.24	41.5 ± 10	44.2 ± 9.1	0.45	40.9 ± 9.7	45.1 ± 9.4	0.21
PCS	mean ± SD	47.6 ± 10.3	49.4 ± 8.2	45.9 ± 11.9	0.22	48.4 ± 9.4	45.5 ± 12.5	0.46	47.5 ± 9.5	47.7 ± 12.3	0.83	49.2 ± 7.3	44.6 ± 14.2	0.33
MCS	mean ± SD	45.0 ± 9.7	49.4 ± 7.6	40.8 ± 9.8	< 0.001	46.9 ± 8.8	40 ± 10.7	0.05	47.2 ± 9	39.7 ± 9.7	0.03	47.9 ± 8.3	39.4 ± 10.1	0.01
RCS	mean ± SD	50.6 ± 11.3	53.8 ± 7.2	47.6 ± 13.7	0.23	51.9 ± 9.4	47.2 ± 15.1	0.43	49.9 ± 12	52.2 ± 9.6	0.62	51.2 ± 10	49.5 ± 13.7	0.96
Threaten of cancer	n (%)	37 (86.0)	18 (85.7)	19 (86.4)	< 0.001	27 (87.1)	10 (83.3)	0.55	25 (83.3)	12 (92.3)	0.40	25 (89.3)	12 (80.0)	0.34
*Menopause*														
Menopause	n (%)	17 (39.5)	7 (33.3)	10 (45.5)	0.31	15 (48.4)	2 8(16.7)	0.06	11 (36.7)	6 (46.2)	0.40	11 (39.3)	6 (40.0)	0.61
Treatment of menopausal symptom	n (%)	5 (11.6)	1 (4.8)	4 (18.2)	0.19	2 (6.5)	3 (25.0)	0.12	4 (13.3)	1 (7.7)	0.52	2 (7.1)	3 (20.0)	0.22
Number of menopausal symptom	mean ± SD	11.3 ± 5.0	8.6 ± 4.0	13.9 ± 4.4	< 0.001	10.2 ± 4.3	14.2 ± 5.6	0.02	10.3 ± 4.3	13.7 ± 5.7	0.04	9.3 ± 3.8	15.1 ± 4.8	< 0.001
Hot flashes of face or upper body	n (%)	23 (53.5)	11 (52.4)	12 (54.5)	0.89	15 (48.4)	8 (66.7)	0.28	18 (60.0)	5 (38.5)	0.19	14 (50.0)	9 (60.0)	0.53
Sweat easily	n (%)	29 (67.4)	14 (66.7)	15 (68.2)	0.92	20 (64.5)	9 (75.0)	0.39	20 (66.7)	9 (69.2)	0.58	17 (60.7)	12 (80.0)	0.17
Unable to fall asleep at night	n (%)	14 (32.6)	7 (33.3)	7 (31.8)	0.92	8 (25.8)	6 (50.0)	0.13	8 (26.7)	6 (46.2)	0.18	6 (21.4)	8 (53.3)	0.04
Fall asleep but often awake at night	n (%)	23 (53.5)	8 (38.1)	15 (68.2)	0.05	13 (41.9)	10 (83.3)	0.01	13 (43.3)	10 (76.9)	0.04	11 (39.3)	12 (80.0)	0.01
Easily excitable, often irritable	n (%)	29 (67.4)	10 (47.6)	19 (86.4)	0.01	19 (61.3)	10 (83.3)	0.15	19 (63.3)	10 (76.9)	0.31	17 (60.7)	12 (80.0)	0.17
Always anxious	n (%)	22 (51.2)	5 (23.8)	17 (77.3)	< 0.001	13 (41.9)	9 (75.0)	0.05	13 (43.3)	9 (69.2)	0.12	10 (35.7)	12 (80.0)	0.01
Worry about minor things	n (%)	26 (60.5)	8 (38.1)	18 (81.8)	< 0.001	18 (58.1)	8 (66.7)	0.44	16 (53.3)	10 (76.9)	0.15	14 (50.0)	12 (80.0)	0.06
Worry and often become depressed	n (%)	23 (53.5)	4 (19.0)	19 (86.4)	< 0.001	14 (45.2)	9 (75.0)	0.08	15 (50.0)	8 (61.5)	0.49	11 (39.3)	12 (80.0)	0.01
Lack of energy, easily tired	n (%)	25 (58.1)	7 (33.3)	18 (81.8)	< 0.001	16 (51.6)	9 (75.0)	0.16	15 (50.0)	10 (76.9)	0.10	12 (42.9)	13 (86.7)	0.01
Tired feeling of eyes	n (%)	38 (88.4)	16 (76.2)	22 (100.0)	0.02	27 (87.1)	11 (91.7)	0.57	25 (83.3)	13 (100.0)	0.15	23 (82.1)	15 (100.0)	0.10
Forgetful	n (%)	29 (67.4)	13 (61.9)	16 (72.7)	0.45	21 (67.7)	8 (66.7)	0.61	19 (63.3)	10 (76.9)	0.31	18 (64.3)	11 (73.3)	0.40
Dizziness	n (%)	17 (39.5)	7 (33.3)	10 (45.5)	0.42	9 (29.0)	8 (66.7)	0.03	11 (36.7)	6 (46.2)	0.56	8 (28.6)	9 (60.0)	0.04
Heart pounds quickly	n (%)	15 (34.9)	3 (14.3)	12 (54.5)	0.01	10 (32.3)	5 (41.7)	0.41	8 (26.7)	7 (53.8)	0.09	6 (21.4)	9 (60.0)	0.01
Tight feeling of chest	n (%)	7 (16.3)	2 (9.5)	5 (22.7)	0.23	3 (9.7)	4 (33.3)	0.08	3 (10.0)	4 (30.8)	0.11	4 (14.3)	3 (20.0)	0.47
Headaches	n (%)	25 (58.1)	9 (42.9)	16 (72.7)	0.05	16 (51.6)	9 (75.0)	0.16	16 (53.3)	9 (69.2)	0.33	13 (46.4)	12 (80.0)	0.03
Shoulder or neck stiffness	n (%)	39 (90.7)	20 (95.2)	19 (86.4)	0.32	29 (93.5)	10 (83.3)	0.31	27 (90.0)	12 (92.3)	0.65	25 (89.3)	14 (93.3)	0.56
Back or low back pain	n (%)	29 (67.4)	13 (61.9)	16 (72.7)	0.45	19 (61.3)	10 (83.3)	0.15	19 (63.3)	10 (76.9)	0.31	17 (60.7)	12 (80.0)	0.17
Joint of hands and feet painful	n (%)	21 (48.8)	9 (42.9)	12 (54.5)	0.44	13 (41.9)	8 (66.7)	0.15	13 (43.3)	8 (61.5)	0.27	11 (39.3)	10 (66.7)	0.09
Coldness	n (%)	28 (65.1)	8 (38.1)	20 (90.9)	< 0.001	19 (61.3)	9 (75.0)	0.32	18 (60.0)	10 (76.9)	0.24	14 (50.0)	14 (93.3)	< 0.001
Numbness of hands and feet	n (%)	14 (32.6)	6 (28.6)	8 (36.4)	0.59	7 (22.6)	7 (58.3)	0.03	8 (26.7)	6 (46.2)	0.18	6 (21.4)	8 (53.3)	0.04
Recently sensitive to sound	n (%)	12 (27.9)	1 (4.8)	11 (50.0)	< 0.001	8 (25.8)	4 (33.3)	0.45	6 (20.0)	6 (46.2)	0.09	4 (14.3)	8 (53.3)	0.01
*Oral symptom*														
Xerostomia	n (%)	22 (51.2)	–	–	–	14 (45.2)	8 (66.7)	0.21	13 (43.3)	9 (69.2)	0.12	9 (32.1)	13 (86.7)	< 0.001
Taste disturbance	n (%)	12 (27.9)	4 (19.0)	8 (36.4)	0.21	–	–	–	6 (20.0)	6 (46.2)	0.09	2 (7.1)	10 (66.7)	< 0.001
Burning mouth	n (%)	13 (30.2)	4 (19.0)	9 (40.9)	0.08	7 (22.6)	6 (50.0)	0.09	–	–	–	2 (7.1)	11 (73.3)	< 0.001
Unstimulated saliva (mL/min)	mean ± SD	0.3 ± 0.2	0.4 ± 0.2	0.2 ± 0.2	< 0.001	0.4 ± 0.2	0.2 ± 0.1	0.02	0.4 ± 0.2	0.2 ± 0.2	0.03	0.4 ± 0.2	0.2 ± 0.2	0.01
Stimulated saliva (g)	mean ± SD	3.4 ± 2.0	3.4 ± 1.5	3.4 ± 2.4	0.87	3.7 ± 2.2	2.9 ± 1.6	0.38	3.6 ± 2.1	3.1 ± 1.9	0.84	3.7 ± 2.2	3 ± 1.8	0.61
TCI	mean ± SD	23.5 ± 14.8	23.8 ± 13.4	23.2 ± 16.3	0.99	26.3 ± 14.1	16.2 ± 14.7	0.04	26.3 ± 14.1	17.1 ± 15	0.04	26.6 ± 12.8	17.8 ± 17	0.05

2OSC, two or more oral sensory complaints; CNS, central nervous system; MCS, mental component summary; PCS, physical component summary; RCS, role/social component summary; TCI, tongue coating index

### Oral sensory complains and QOL

Scores on the mental component summary of the QOL questionnaire were significantly lower in women with xerostomia (*p* < 0.001), burning mouth (*p* = 0.03) and 2OSC (*p* = 0.01) than in the other participants.

### Oral sensory complains and menopausal symptoms

Women with xerostomia (*p* < 0.001), taste disturbance (*p* = 0.02) burning mouth (*p* = 0.04) and 2OSC (*p* < 0.001) had significantly more menopausal symptoms than did the other participants.

### Oral sensory complains and saliva volume

Unstimulated saliva volume was significantly smaller in participants who had xerostomia (*p* < 0.001), taste disturbance (*p* = 0.02) burning mouth (*p* = 0.03) and 2OSC (*p* = 0.01) than in those who did not.

### Factors associated with oral symptoms

As shown in Table 2, logistic regression analysis showed associations between the number of menopausal symptoms and xerostomia, taste disturbance, burning mouth and 2OSC. Number of menopausal symptom was to be an explanatory variable for xerostomia (odds ratio 1.331), taste disturbance (odds ratio 1.204), burning mouth (odds ratio 1.162) and 2OSC (odds ratio 1.358) with statistical significantly difference. The discriminant probability was 69.8% for xerostomia, 81.4% for taste disturbance, 78.6% for burning sensation and 85.7% for 2OSC (*p* < 0.001).

**Table 2:** Factors associated with xerostomia, taste disturbance, burning mouth and two or more oral sensory complaints (logistic regression analysis)

Objective variable	Explanatory variable	Odds ratio	95% confidence interval	*P*
Xerostomia	Number of menopausal symptoms	1.331	1.112–1.592	0.002
Taste disturbance	Number of menopausal symptoms	1.204	1.026–1.413	0.023
Burning mouth	Number of menopausal symptoms	1.162	1.002–1.347	0.048
2OSC	Number of menopausal symptoms	1.358	1.121–1.646	0.002

2OSC, two or more oral sensory complaints

## Discussion

To the best of our knowledge, this is a first reported study to investigate the factors associated with xerostomia, taste disturbance, burning mouth and 2OSC in perimenopausal women simultaneously. Logistic regression analysis showed associations between the number of menopausal symptoms and xerostomia, taste disturbance, burning mouth and 2OSC. Moreover, unstimulated saliva volume was associated with these symptoms according to univariate analysis. The basic cause of menopausal symptoms is the complex relationship of estrogen metabolism and the autonomic nervous system [16]. Salivary secretion is regulated by the autonomic nervous system and unstimulated saliva volume was smaller in participants with menopausal symptoms in the present study. Sensations of coldness result from contraction of peripheral blood vessels, which may also be related to autonomic nervous function. The parasympathetic nervous system is reportedly less active in individuals who are sensitive to cold than in healthy people who are not [17]. It has been suggested that peripheral circulatory hypofunction is caused by excessive sympathetic nerve activity [17]. Evaluation of autonomic nervous system function is required in future.

Univariate analysis showed that participants with taste disturbance and 2OSC were taking significantly more medications than the other participants. Recently, polypharmacy was identified as an issue in older individuals because it is associated with frailty [18] and falls [19]. Polypharmacy may also be an issue in perimenopausal women. Agents targeting the central nervous system were more frequently taken by participants with burning mouth and 2OSC than by those without these characteristics. However, it is difficult to identify the adverse effects of medications because these symptoms can also occur in people with mental health concerns.

Univariate analysis also showed that participants with taste disturbance and burning mouth had a significantly better TCI than those who did not. There are two possible explanations for this finding. One is that these participants’ tongue papillae were too atrophied to allow development of a tongue coating. Atrophy of tongue papillae may cause taste disturbance or a burning sensation. The other possible explanation is tongue cleaning. Participants who experience taste distortion or abnormal sensations in their tongues may tend to clean them with tongue brushes more frequently. In this study, we did not investigate the state of tongue papillae or frequency of tongue cleaning.

This study had some limitations. The symptoms investigated were only assessed on the basis of participants’ subjective experiences; no diagnoses were made. We did not determine taste thresholds or do tests to exclude tongue cancer and other tongue disorders. Additionally, we did not use an accelerometer to evaluate autonomic nervous system function objectively [20]. Evaluation of autonomic nervous system function and measurement of the blood flow in the oral mucosa is required to further investigate these possibilities. Furthermore, we did not check serum estrogen concentrations. Some participants reported xerostomia but had normal saliva volumes. That is, xerostomia and hyposalivation did not invariably coexist. Although QOL is impacted by subjective sensations, not objective diagnoses, investigation of objective factors is needed to clarify the relationship between these symptoms and menopause.

In conclusion, we found associations between the number of menopausal symptoms and xerostomia, taste disturbance, burning mouth and 2OSC in our cohort of perimenopausal women. Effective management of all of these symptoms may decrease the incidence and severity of oral discomfort, improving the QOL of perimenopausal women.

## Supplementary Information

**Additional file 1**. Supplementary table 1.

**Additional file 2**. Supplementary table 2.

## Data Availability

All data generated or analysed during this study are included in this published article.
