# Supplementary material for: Menopausal symptoms are associated with oral sensory complaints in perimenopausal women: an observational study
Source: BMC Womens Health. 2021 Jun 30;21:262. doi: 10.1186/s12905-021-01401-6 (PMC8243452; doi:10.1186/s12905-021-01401-6)
Supplement: Supplementary file 1 — Additional file 1. Supplementary table 1. [file 12905_2021_1401_MOESM1_ESM.docx]

Supplementary Table 1

| Characteristics of participants | | |
| --- | --- | --- |
|  | Age | years old |
|  | Height | cm |
|  | Body weight | kg |
|  | Marriage | yes / no |
|  | Children | yes / no |
|  | Job | yes / no |
|  | Smoking | yes / no |
|  | Medical history |  |
|  | Medication |  |
|  | Menstrual status | yes / no |
|  | History of treatment for menopausal symptoms, | yes / no |
|  | Sleep duration | min |
|  | Quality of sleep | good / bad |
| Oral symptoms | | |
|  | Xerostomia | yes / no |
|  | Taste disturbance | yes / no |
|  | Burning mouth, | yes / no |
|  | Threaten of oral cancer | yes / no |
