# Supplementary material for: Menopausal symptoms are associated with oral sensory complaints in perimenopausal women: an observational study
Source: BMC Womens Health. 2021 Jun 30;21:262. doi: 10.1186/s12905-021-01401-6 (PMC8243452; doi:10.1186/s12905-021-01401-6)
Supplement: Supplementary file 2 — Additional file 2. Supplementary table 2. [file 12905_2021_1401_MOESM2_ESM.docx]

Supplementary Table２

| Hot flashes of face or upper body |
| --- |
| Sweat easily |
| Unable to fall asleep at night |
| Fall asleep but often awake at night |
| Easily excitable, often irritable |
| Always anxious |
| Worry about minor things |
| Worry and often become depressed |
| Lack of energy, easily tired |
| Tired feeling of eyes |
| Forgetful |
| Dizziness |
| Heart pounds quickly |
| Tight feeling of chest |
| Headaches |
| Shoulder or neck stiffness |
| Back or low back pain |
| Joint of hands and feet painful |
| Coldness |
| Numbness of hands and feet |
| Recently sensitive to sound |
